# Mechanical Properties of Concrete Mixes with Selectively Crushed Wind Turbine Blade: Comparison with Raw-Crushing

**DOI:** 10.3390/ma17246299

**Published:** 2024-12-23

**Authors:** Víctor Revilla-Cuesta, Ana B. Espinosa, Roberto Serrano-López, Marta Skaf, Juan M. Manso

**Affiliations:** 1 Department of Civil Engineering, University of Burgos, 09001 Burgos, Spain; vrevilla@ubu.es (V.R.-C.); robertosl@ubu.es (R.S.-L.); jmmanso@ubu.es (J.M.M.); 2 Department of Construction, University of Burgos, 09001 Burgos, Spain; aespinosa@ubu.es

**Keywords:** selectively crushed wind turbine blade, concrete, mechanical performance, significative effect, raw-crushing, resulting material comparison

## Abstract

The glass fiber-reinforced polymer (GFRP) materials of wind turbine blades can be recovered and recycled by crushing, thereby solving one of the most perplexing problems facing the wind energy sector. This process yields selectively crushed wind turbine blade (SCWTB), a novel waste that is almost exclusively composed of GFRP composite fibers that can be revalued in terms of their use as a raw material in concrete production. In this research, the fresh and mechanical performance of concrete made with 1.5%, 3.0%, 4.5%, and 6.0% SCWTB is studied. Once incorporated into concrete mixes, SCWTB waste slightly reduced slumps due to the large specific surface area of the fibers, and the stitching effect of the fibers on mechanical behavior was generally adequate, as scanning electron microscopy demonstrated good fiber adhesion within the cementitious matrix. Thus, despite the increase in the content of water and plasticizers when adding this waste to preserve workability, the compressive strength only decreased in the long term with the addition of 6.0% SCWTB, a value of 45 MPa always being reached at 28 days; Poisson’s coefficient remained constant from 3.0% SCWTB; splitting tensile strength was maintained at around 4.7 MPa up to additions of 3.0% SCWTB; and the flexural strength of mixes containing 6.0% and 1.5% SCWTB was statistically equal, with a value near 6.1 MPa. Furthermore, all mechanical properties of the concrete except for flexural strength were improved with additions of SCWTB compared to raw crushed wind turbine blade, which apart from GFRP composite fibers contains approximately spherical polymer and balsa wood particles. Flexural strength was conditioned by the proportion of fibers, their dimensions, and their strength, which were almost identical for both waste types. SCWTB would be preferable for applications in which compression stresses predominate.

## 1. Introduction

Fiber-reinforced polymer (FRP) is a composite formed of two components: a polymeric matrix and fibers [1]. The polymeric matrix is usually a stiffening epoxy resin, within which the fibers are embedded to add strength [2]. The most common fibers are usually either glass or carbon, producing glass fiber-reinforced polymer (GFRP) [3] and carbon fiber-reinforced polymer (CFRP) [4,5], respectively. FRP density, between 1.8 and 2.0 kg/dm^3^ [1], is not very high, and at the same time, FRP shows very good structural behavior [6], especially for bending tensile conditions in the fiber direction [7]. In addition, it can be hot-molded into a wide variety of shapes during manufacture, in the same way as conventional polymers [8]. Its low density and moldability offer advantages over traditional structural materials such as concrete and steel [6], which has led to an increasing number of applications. For example, FRP can be used in structural profiles [6], in concrete reinforcement bars that replace steel bars to prevent corrosion [7], and even as external reinforcement for the rehabilitation of concrete structures [3]. Nevertheless, FRP is most commonly used in the field of mechanical and aeronautical engineering for the design of high-mechanical-strength and low-weight components [4,9].

Notwithstanding, FRP also presents disadvantages, its recycling and revaluation at its useful-life end being a major challenge in this field [10]. Once decommissioned, the most widespread practice is to bury FRP at landfill sites, which entails high environmental impacts and land occupation [11,12]. Seeking alternatives to avoid landfilling, two main types of recycling procedures have been addressed in the scientific research, although their development and optimization are still in progress:

- First, there are chemical processes, in which the epoxy resin matrix is removed to recover the fibers for new uses [10,13,14]. Epoxy resin is thermoset, so it does not melt when heated [2]. It is therefore necessary to burn it at high temperature [13], in a process known as pyrolysis [15], or to dissolve it by applying chemicals, in a process known as solvolysis [10]. Both processes generate gases and toxic products [14]; both require high energy consumption and are therefore very costly [16]; in addition, the characteristics of the resulting fibers are poorer than those of the original ones, especially in terms of tensile strength and surface adherence [2,17].
- The second possibility is mechanical treatment, which consists of machining the FRP [18] or crushing and sieving it [16,19]. In this way, FRP elements of varying shapes and forms can be produced, for which new uses can be found [18,20]. The epoxy resin matrix is not removed during the mechanical treatment, so those elements consist of the matrix and either glass or carbon fibers [1]. FRP composite fibers are usually produced in those processes [20,21].

Wind turbines have a service life of between 25 and 30 years, at which point they are no longer economically viable and need to be dismantled or repowered, i.e., replaced by others of greater size and power [22]. Currently, the first wind turbines installed in the last years of the 20th century are almost at their useful-life end, and increasing numbers of wind turbines will soon have to be decommissioned [23,24]. It has been suggested in some estimates that half of all installed wind power capacity in countries such as Spain, the fifth largest global wind energy producer, will have to be decommissioned by 2030 [25]. The key problematic fact in this process is that GFRP, which is the principal constituent of wind turbine blades [26], has no widely accepted recycling process [27,28], so its environmental impact can only increase [12]. There are scientific studies on both chemical and mechanical treatments for the GFRP found in wind turbine blades [29]. Among the products of mechanical treatment, the addition of the yielded material into concrete as either filler, aggregate, or fiber has been analyzed [18,20,21,30]. GFRP filler considerably increases the porosity of concrete matrices, which significantly worsens the mechanical performance of concrete [30], and low adhesion within the cementitious matrix of the GFRP-derived aggregate results in premature failure of the concrete elements due to slippage and debonding [18]. These phenomena could also affect other properties of concrete, such as workability [31] or fire resistance [32], as is the case with other wastes. Thus, the most widespread solution is the production of fiber-reinforced concrete through the addition of GFRP composite fibers, as those fibers show adequate adhesion within the cementitious matrix and have a stitching effect [20]. They are therefore able to increase concrete toughness and ductility, as well as its load-bearing capacity in a similar way to conventional steel fibers [21,33].

Another problem of wind turbine blades is that they not only consist of GFRP, but also of other components as varied in nature as balsa wood, which has a density of around 0.2 kg/dm^3^ [34] and which reduces the weight of the blade in areas where the mechanical stress is not high [15]; polymers, which are mainly used to form the internal stiffeners of the blades and to glue the joints between materials [26]; and external coating gels that protect the blades from aggressive atmospheric agents [14]. Exclusively mechanical treatments of GFRP, if previously separated from the other blade components by cutting, can produce GFRP composite fibers to add to concrete [21]. However, it is also very necessary to look for ways to recycle its other components [20]. In view of this situation, researchers at the University of Burgos, Spain have proposed the non-selective cutting and crushing of wind turbine blades, obtaining a material that they have called raw crushed wind turbine blade (RCWTB) [35]. This product is composed of GFRP composite fibers, but also of approximately spherical polymer and balsa wood particles that act as a lightweight aggregate and can be used as a raw material in concrete production [35,36]. The authors demonstrated that RCWTB, added in quantities of up to 6.0% to concrete mixes, can yield adequate mechanical behavior, i.e., 28-day compressive strengths of over 40 MPa [37]. In addition, higher RCWTB contents even enable slightly increased concrete flexural strengths, due to the stitching effects within the cementitious matrix of the GFRP composite fibers [37].

In this paper, the mechanical performance of concrete containing selectively crushed wind turbine blade (SCWTB) is evaluated, which is the first objective of this study. SCWTB is a novel type of waste from wind turbine blades composed almost exclusively of crushed GFRP following separation by cutting from the other blade constituents. Thus, this material is almost exclusively formed of GFRP composite fibers, containing minimal amounts of balsa wood and polymers. The GFRP composite fibers in SCWTB showed practically identical characteristics to those of the RCWTB from the previous investigation [35]. In addition, the mix design of the concrete mixes with SCWTB was identical to the previously developed mix design for RCWTB [37], but with SCWTB additions of 1.5%, 3.0%, 4.5%, and 6.0% instead of RCWTB. Therefore, the mechanical behavior of concrete containing SCWTB may not only be analyzed in this study, but it may also be compared with the behavior of concrete containing RCWTB, which is the second objective of this research. In this way, the aim is to elucidate, according to the mechanical performance of concrete, which is the most suitable procedure for mechanically treating wind turbine blades. Thus, the type of by-product from wind turbine blades that is more adequate for use in concrete can be identified from a mechanical viewpoint. The final target of this research line is to produce an added-value product that can be successfully used in concrete manufacturing as a raw material while reducing the consumption of natural resources in concrete production. The two objectives of this study, described in this paragraph, are novel in essence within the scientific literature and have not been addressed so far in the research related to concrete science according to the author’s knowledge.

## 2. Materials and Methods

### 2.1. Concrete Components

#### 2.1.1. Conventional Components

Regarding conventional components, the mixes in this research were composed of CEM II/A-L 42.5 R cement, whose content of Portland clinker was between 80% and 94% per EN 197-1 [38]; tap water; two plasticizers to increase concrete strength, because of the decrease in water demand that they entailed [39]; and three fractions of siliceous aggregate: 0/2 mm, 2/6 mm, and 6/22 mm. All the aggregates presented the usual values for their physical properties [40], with a density between 2.58 kg/dm^3^ and 2.62 kg/dm^3^, and water absorption levels over 24 h between 0.52% wt. and 1.83% wt.

#### 2.1.2. Selectively Crushed Wind Turbine Blade (SCWTB)

SCWTB was produced following a mechanical production process that was similar to the production of RCWTB [35]. The panels from the walls of wind turbine blades selected to obtain SCWTB were of an approximately rectangular shape with a side length of between 20 cm and 30 cm. As the SCWTB had to be almost exclusively composed of GFRP, GFRP was separated from the other blade components by mechanical cutting with a jig-saw. Subsequently, the panels were knife-milled, similar to the models used for crushing plastic containers [41], and then sieved with a 10-mm aperture sieve. Those elements that were retained were crushed and sieved again. All the blade panels were continuously crushed and sieved in the same facilities and during the same period of time, which ensured the uniformity of the obtained SCWTB throughout all the concrete mixes.

**Figure 1:**
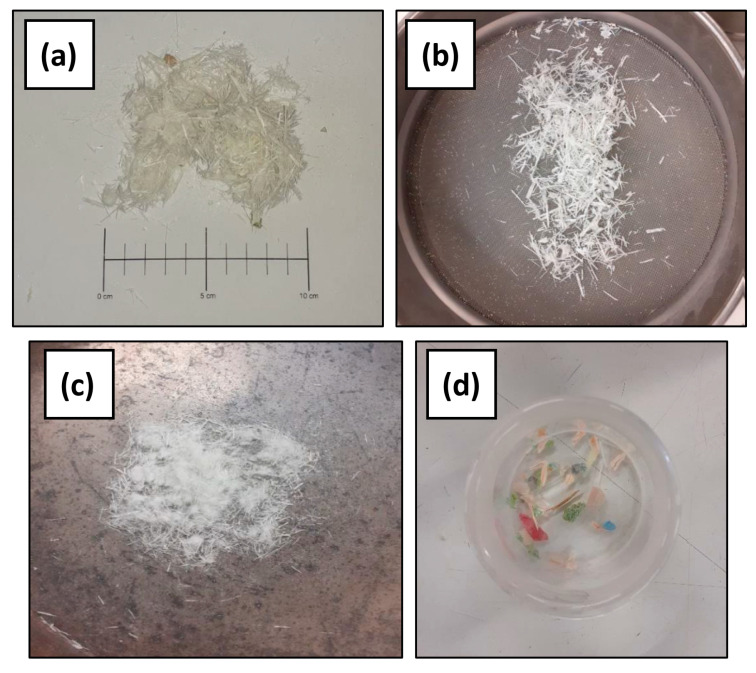
Selectively crushed wind turbine blade: (**a**) resulting material; (**b**) fibers; (**c**) micro-fibers; (**d**) other components (balsa wood, polyurethane foam, and other polymers).

The resulting SCWTB is illustrated in Figure 1a and its physical properties, which were measured in three SCWTB samples obtained through quartering to guarantee that the results were representative of the whole batch of SCWTB, are detailed in Table 1. This waste mainly consists of fibers (Figure 1b), defined as GFRP composite fibers retained by a 1-mm aperture sieve, which presented dimensions and tensile strengths (last three rows of Table 1) similar to some commercial synthetic fibers [42,43]. Micro-fibers (Figure 1c) were the second major component, which basically consisted of very fine GFRP composite fibers and glass fibers separated from the epoxy resin matrix that passed through the 1-mm sieve and sometimes agglomerated in the form of “fluff”. Minimal amounts of other components such as balsa wood, polyurethane foam, and other polymers were also present (Figure 1d). According to the standard deviations obtained for the proportions of the SCWTB components listed in Table 1, it can be stated that GFRP, balsa wood, and polymers were consistently separated during the SCWTB production process. Furthermore, 98.1% wt. of the SCWTB was GFRP, which demonstrated the great efficiency reached in recovering this material during this process. However, all these aspects cannot be fully verified until SCWTB is produced at an industrial level, i.e., in large quantities, an aspect that can be addressed in future research.

The overall material presented a density of 1.50 kg/dm^3^, a lower result than that of commercial fibers [42,43], and a high sponginess without compaction [45], with an apparent density of only 84.3 kg/m^3^.

### 2.2. Mix Design

Four mixes were prepared with SCWTB contents of 1.5%, 3.0%, 4.5%, and 6.0% by volume, respectively. The mixes were labelled with the letters MS followed by the SCWTB amount, so that, for example, the MS4.5 mix was the mix containing 4.5% SCWTB. The design of the concrete mixtures, which is shown in Table 2, was identical to that of the previous study with RCWTB [37], but with a different type of wind turbine blade waste added. Thus, the aspects considered in the mix design were:

- The proportions of water, cement, and aggregates were initially set on the basis of regulations [40] and subsequently by empirical testing. The mix with the lowest SCWTB content was designed to have a slump of between 10.0 and 15.0 cm, i.e., a S3 slump class per EN 206 [38].
- The amounts of the aggregate fractions were defined by an optimal fitting to the Fuller curve with a maximum aggregate size of 22 mm. Thus, 45.1% of the whole volume of aggregate was sized at 6/22 mm, 30.1% 2/6 mm, and 24.8% 0/2 mm.
- SCWTB was added to concrete with no substitution of any other component. The addition of that waste therefore increased the volume of the concrete, which resulted in a decrease in cement content per cubic meter, as shown under the column headings “comparative” and “kg/m^3^” of Table 2, a design that also increased concrete sustainability [46].
- The proportion of water and plasticizers was increased when adding SCWTB at the same rate as for RCWTB [35]. The objective was to maintain an S3 slump class according to EN 206 [38].
- The concrete mechanical performance with 0.0% and 1.5% RCWTB was equivalent according to previous studies [35,37]. Thus, the minimum SCWTB content considered in this research was 1.5%, as the mechanical performance of concrete containing 1.5% SCWTB would be equivalent to that of conventional concrete. The quantities of sustainable raw materials within the concrete were also maximized in this way, and the comparison of the effect of both wastes from wind turbine blades in the mechanical performance of concrete was simplified

### 2.3. Experimental Plan

The concrete mixes were prepared in this research following the same procedure as for the RCWTB mixtures, so that the preparation method had a similar effect on concrete behavior, facilitating comparison between both waste types [37]. The components were therefore added in five different times to a vertical axis mixer: first, aggregates and 30% water; second, cement and 70% water; third, half of the plasticizers in 0.25 L of water; fourth, SCWTB; and fifth, the rest of the plasticizers in 0.25 L of water. After each stage, concrete was mixed for three, three, two, two, and five minutes, respectively, as detailed in previous studies [35]. The objective was to ensure maximum concrete workability and strength by aggregate saturation, adequate cement hydration, and optimal distribution of SCWTB in the concrete mass [47,48].

**Figure 2:**
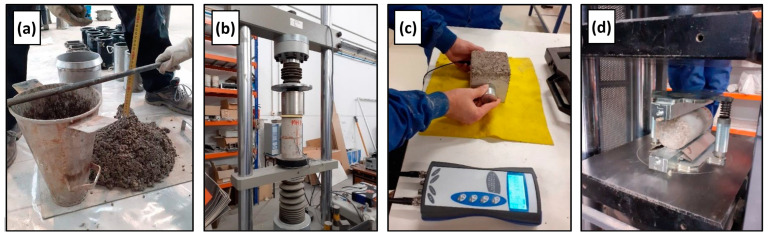
Illustration of some of the tests conducted: (**a**) slump test; (**b**) modulus of elasticity and Poisson’s coefficient; (**c**) UPV; (**d**) splitting tensile strength.

Once mixing was completed, the fresh behavior was evaluated, and the specimens for the hardened state tests were manufactured. All the specimens were stored in a humid chamber, in accordance with EN 12390-2 (temperature of 20 ± 2 °C and moisture of 90 ± 5%) [38] until each testing age. During all tests at each stage, the international standards were followed, and the specimen type and dimensions are listed in Table 3. Some of these tests are illustrated in Figure 2. The hardened state experimental plan conducted was aimed at evaluating the effect of the addition of SCWTB and the type of wind turbine blade waste on all the dimensions of the mechanical behavior of concrete. All the strength tests were performed with a universal concrete strength-testing press with a load capacity of up to 300 tons.

Based on the authors’ previous experience [37], three specimens per hardened property and mix were initially tested, thus obtaining three experimental results for each one of them. Outliers were detected among these three experimental results (Grubbs’ test by comparison with reference values) and as many new specimens were tested as outliers found. This process continued until three experimental results were identified as non-outliers. In this way, the standard deviation among all the mixtures for the same property was similar, minimizing the experimental error and statistically guaranteeing the validity of the trends obtained. The final result of each test for each mix was calculated as the arithmetic mean of the individual values of the three specimens identified as non-outliers, which are detailed throughout the article jointly with their standard deviations.

The study also included a scanning electron microscopy (SEM) micro-structural analysis of the interfacial transition zone in MS6.0 mixture fragments, examining the GFRP composite fibers of the SCWTB within the cementitious matrix at 90 days. A JEOL JSM-6460LV microscope (Peabody, MA, USA)was used for this analysis. The relations between concrete properties at 28 days were also analyzed; the significance of the SCWTB content on each concrete property was evaluated through an analysis of variance (ANOVA) at a confidence level of 95%, which also allowed for the verification of the validity of the SCWTB contents considered in this research to accurately and reliably define the effect of SCWTB in the mechanical performance of concrete; the significance of the type of wind turbine blade waste, RCWTB, or SCWTB on each mechanical property of concrete was also explored, also with the support of an ANOVA.

## 3. Results and Discussion

### 3.1. Fresh Performance

The results of the three in-fresh tests are shown in Table 4. Both slump and fresh density slightly decreased with the addition of SCWTB, while the air content rose slightly:

- The slump decreased by 4 cm (26.7%) following an increase in SCWTB content from 1.5% to 6.0%. Higher amounts of SCWTB in the concrete mix increased the fiber surface area that the water had to cover, which in turn reduced the proportion of free water [21]. Moreover, the fibers were of an irregular shape, increasing the internal friction and hindering the flow of the other components [48]. However, adjustments to the water and plasticizer content eventually yielded an S3 slump class following EN 206 [38].
- The fresh density decreased slightly, by 3.8%, between the mixes with higher and lower SCWTB contents. SCWTB presented a lower density than the rest of the concrete components (Table 1), which caused this reduction along with the increase in water and admixtures [49].
- Finally, the air content remained almost constant with only a minimal increase. The irregular shape of the GFRP composite fibers may have led to a small increase in air bubbles trapped in the fresh concrete [48].

### 3.2. Hardened Density

**Figure 3:**
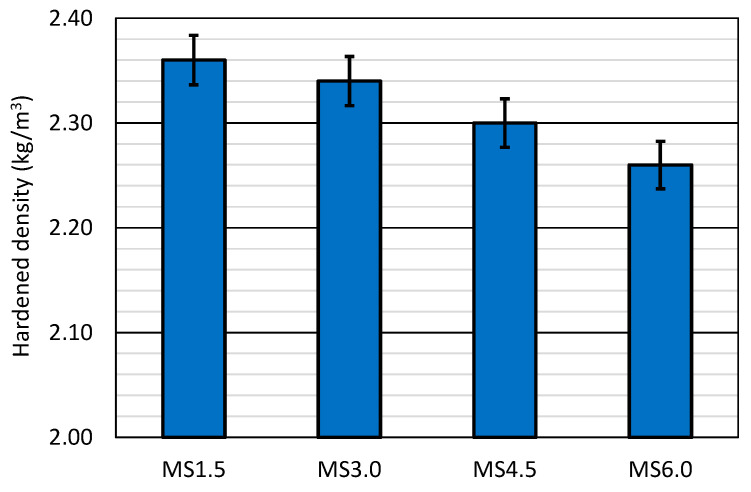
Hardened density of the concrete mixes.

The hardened (Figure 3) and fresh densities of the mixes followed the same trend, so this property was reduced in an approximately linear way with increasing SCWTB contents. The decrease in hardened density between the MS1.5 and the MS6.0 mixes was 4.2%, slightly higher than that of fresh density, which demonstrated that the loss in density between the fresh and hardened states increased with additions of SCWTB. This performance was once again caused by the lower density of SCWTB compared to the other concrete components and the augment in water and plasticizers with the addition of that waste [37,49], although SCWTB could also be the cause of an increase in porosity that also favored those results [30,50]. Nevertheless, the density value remained in all cases within the usual range for conventional mass concrete [40].

### 3.3. Compressive Strength

**Figure 4:**
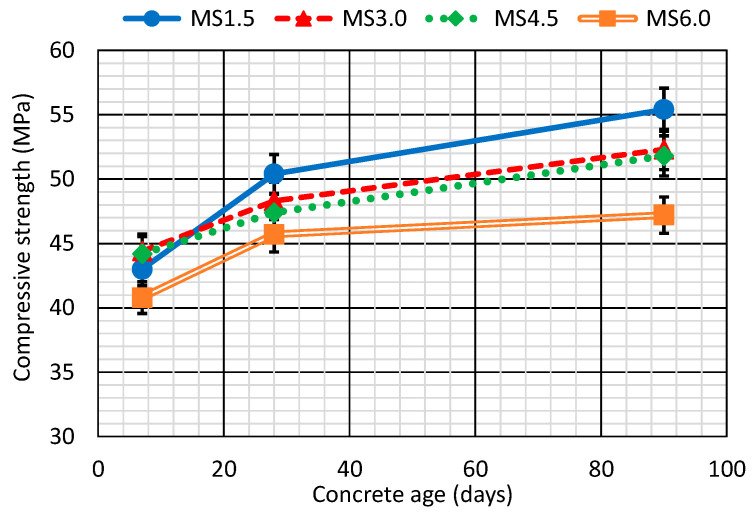
Compressive strength of the concrete mixes.

Figure 4 depicts the compressive strength at 7, 28, and 90 days, which all displayed compressive strengths of over 40 MPa at 7 days and higher than 45 MPa from 28 days onwards. Results that underline that concrete of optimum strength valid for structural applications may be produced using SCWTB according to design standards of concrete structures [40].

Regarding the effects of SCWTB, its increased content in the concrete reduced the 28- and 90-day compressive strength. Thus, the MS1.5 mix had the highest strength at both times and the MS6.0 mix the lowest, with values 9% and 15% lower at 28 and 90 days, respectively. This decrease in strength was due to the increasing water/cement ratio in concrete so as not to lose workability with the incorporation of SCWTB [48], which was so high for large SCWTB contents that it could not be effectively compensated for by the GFRP composite fiber stitching effect within the cementitious matrix [20]. However, that stitching effect was more effective for intermediate SCWTB contents, as found in other research on fiber-reinforced concrete [51], making the decrease in compressive strength lower than expected, especially for the MS4.5 mix. It can be observed that the mixes with 3.0% and 4.5% SCWTB presented very similar compressive strengths at both times, and always approximately 3 MPa lower than the strength of the MS1.5 mix. That beneficial interaction of the GFRP composite fibers at intermediate quantities of SCWTB was especially noticeable at the early times due to the higher deformability of the cementitious matrix at such times, which facilitated the entry of the fibers under load and caused their stitching effect to be more effective [51]. The 7-day compressive strengths of the MS3.0 and the MS4.5 mixes were therefore around 1 MPa higher than that of the MS1.5 mix, despite their higher water/cement ratio. This phenomenon may reflect the efficiency of the GFRP composite fibers in stitching the concrete matrix at an early age, when the matrix is weaker and has not yet developed high strength [42,43].

The use of SCWTB and the resulting increase in the water/cement ratio did not noticeably affect strength development in the short term. Thus, all the mixes with SCWTB contents equal to or greater than 3.0% experienced a strength increase of around 3–5 MPa from 7 to 28 days. The exception was the MS1.5 mix, in which case, this increase was 7.5 MPa, possibly due to the more effective improvement of fiber adhesion within the cementitious matrix over time after low additions of fibers [52]. In the long term, the retarding effect on strength development caused by the increased water/cement ratio was more noticeable, so that the MS1.5, MS3.0, and MS4.5 mixes experienced an increase in strength of around 4–5 MPa from 28 to 90 days, while the strength of the MS6.0 mix only increased by 1 MPa. That performance could also be due to the lower efficiency of the fibers while stitching the cementitious matrix after high additions of SCWTB [51], an aspect previously discussed above.

### 3.4. Elastic Properties

**Figure 5:**
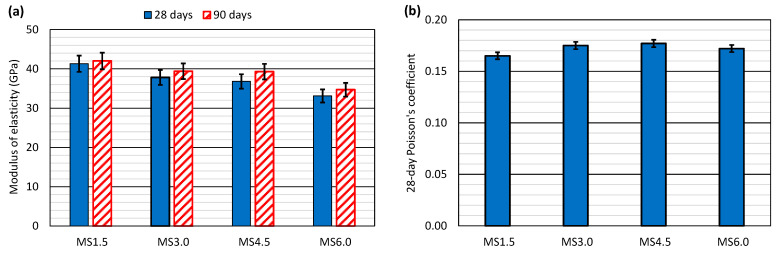
Elastic properties of the concrete mixes: (**a**) modulus of elasticity; (**b**) Poisson’s coefficient.

The elastic properties of the different concretes are displayed in Figure 5. The elastic modulus (Figure 5a) was measured at 28 and 90 days, always reaching values higher than 30 MPa, a value usually accepted as adequate for structural concrete [40,43]. Poisson’s coefficient (Figure 5b), only determined at 28 days, clearly presented values below 0.20, a value normally used as an approximation for all concrete types [40].

The compressive strength (Figure 4) and the modulus of elasticity (Figure 5a) followed very similar graphic curves. Therefore, that property decreased with additions of SCWTB. The MS1.5 mix therefore yielded moduli of 41.3 GPa and 42.0 GPa at 28 and 90 days, respectively, while the elastic moduli of the MS6.0 mix were 33.1 GPa and 34.7 GPa at the same ages. The respective decreases were 17–20%, caused by increasing the water/cement ratio of the mix to preserve workability [48], which was coupled with the GFRP composite fibers not working effectively in the direction perpendicular to the load [33,52]. However, a slight fiber stitching effect was observed in the MS4.5 mix, which shared very similar modulus of elasticity with the MS3.0 mix at both ages, with differences between them of less than 1 GPa. As in the case of compressive strength, the effects of the GFRP composite fibers were more noticeable at intermediate SCWTB contents, as reported in the literature [51]. Finally, in no case did SCWTB affect the development of longitudinal elastic stiffness, so that all the concrete mixtures underwent an increase in their moduli of elasticity of 1–2 GPa between 28 and 90 days.

The stitching effect of the cementitious matrix by the GFRP composite fibers was most noticeable in the direction transverse to the load, as the measurements of the Poisson’s coefficient revealed (Figure 5b). This performance was because the fibers worked in tension in that direction [53], effectively limiting the deformability of the concrete specimens [51]. Thus, the MS1.5 mix presented a Poisson’s coefficient of 0.165, which was increased by 6%, due to the required increment of the water/cement ratio when adding 3.0% SCWTB to the concrete. However, the Poisson’s coefficient remained almost constant at that and higher SCWTB contents, and even decreased by 1.7% between the MS3.0 and MS6.0 mixes.

### 3.5. Ultrasonic Pulse Velocity (UPV)

**Figure 6:**
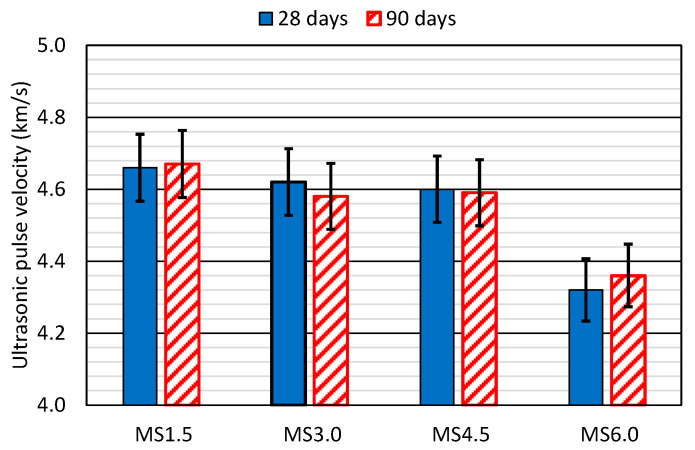
Ultrasonic pulse velocity of the concrete mixes.

The UPV values for all the mixes at both 28 and 90 days are detailed in Figure 6. They were between 4.3 and 4.7 km/s in all cases, which corresponds to good-quality concretes [54]. However, the high dispersion of this non-destructive measurement should be noted [55], which resulted in the large variability of the experimental results, as evidenced from the error bars.

The incorporation of SCWTB between 1.5% and 4.5% resulted in a minimal decrease in UPV, which remained approximately constant between both times. Thus, there was a reduction in UPV of 0.06 km/s at 28 days and 0.08 km/s at 90 days between the MS1.5 and MS4.5 mixes. The higher waste content led to a decrease in both the hardened density of concrete (Figure 3) and its modulus of elasticity (Figure 5a). Both aspects, along with their porosity and the UPV reading [55,56], explain that decrease. However, the reduction was very small, possibly because the GFRP composite fibers present in the SCWTB favored the propagation of the ultrasonic wave in the concrete mass [48]. The MS6.0 mix experienced a more pronounced drop in UPV (0.28 km/s with respect to the MS4.5 mix at 28 days and 0.23 km/s at 90 days), which can be explained by the more notable decrease of its modulus of elasticity, due to the increased water/cement ratio [42].

### 3.6. Bending Tensile Properties

**Figure 7:**
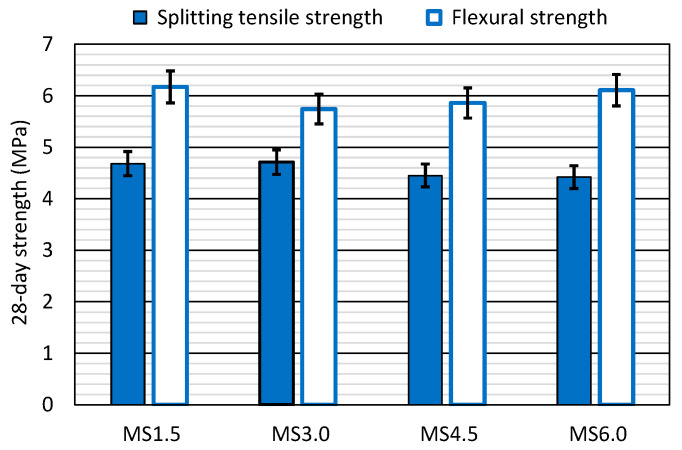
The 28-day bending tensile properties of the concrete mixes.

The bending tensile behavior was characterized by determining the 28-day splitting tensile and flexural strengths, whose results are depicted in Figure 7. The values in all the mixtures indicated they were valid for structural application in which high bending stresses are applied [40]. The flexural strength was 1–1.5 MPa higher than the splitting tensile strength because of the influence of the compressed zone, as is usual in concrete performance [47,51]. Both strengths varied minimally after adding this waste.

The splitting tensile strength decreased with increasing SCWTB contents, although the decrease was not steady. Two groups of concrete mixes could be distinguished. On the one hand were the MS1.5 and the MS3.0 mixes, with splitting tensile strengths of approximately 4.70 MPa. On the other hand were the MS4.5 and the MS6.0 mixtures, with splitting tensile strengths of around 4.45 MPa. That behavior was already obtained in previous research on the addition of RCWTB to concrete [37], and was attributed to the fact that the GFRP composite fibers contained in that residue and their stitching effect were able to compensate for the negative effect of the increase in the amount of water up to a waste content of 3.0% [57]. Above that amount, any increase in the water content had to be so large that the increasing content of fibers could not compensate for the decrease in splitting tensile strength as the water content rose [58]. An identical behavior was observed for RCWTB, with the fibers effectively bridging the cementitious matrix up to additions of 3.0% RCWTB and then the negative implication of the increased water/cement ratio became predominant [57,58].

In relation to flexural strength, there was a loss of 7.0% in flexural strength when the amount of SCWTB was increased from 1.5% to 3.0%. From that point onwards, the strength increased again, with the MS6.0 mix demonstrating a flexural strength only 0.06 MPa lower than the strength of the MS1.5 mix, which had a value of 6.17 MPa. The performance of concrete with RCWTB was similar [37], which demonstrates the validity of both waste types to produce concrete with a high flexural strength when added in high contents [21]. Furthermore, it shows that, despite the behavior of the splitting tensile strength, the flexural strength was the mechanical property that benefited most from the fiber stitching effect within the cementitious matrix, as foreseen, based on other studies on fiber-reinforced concrete [51,52,57]. It was due to the contribution of the compressed zone within the concrete specimens that could withstand bending stresses, which meant that the fibers could act more effectively to compensate for the reduction in strength derived from the increase in the water/cement ratio [48].

### 3.7. Relationship Between Properties

**Figure 8:**
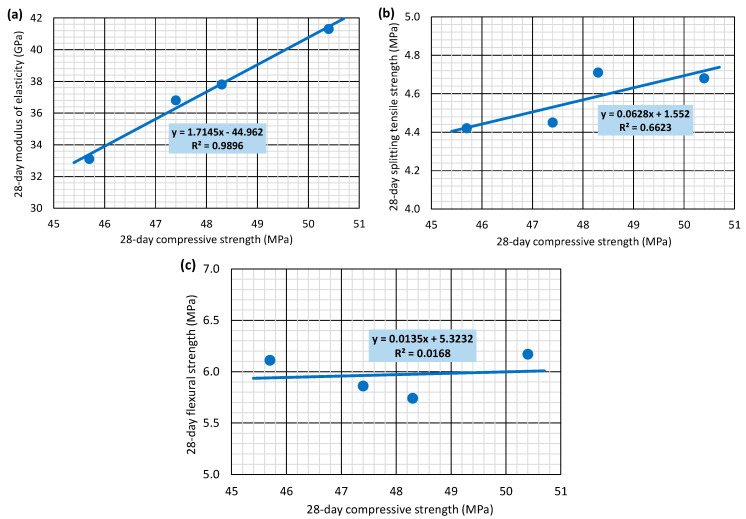
Relationship between 28-day compressive strength and (**a**) 28-day modulus of elasticity; (**b**) 28-day splitting tensile strength; (**c**) 28-day flexural strength.

**Figure 9:**
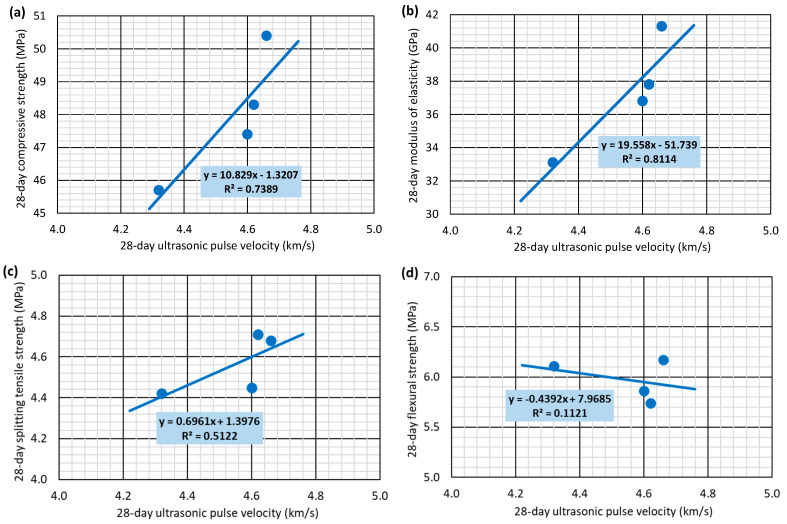
Relationship between 28-day ultrasonic pulse velocity and: (**a**) 28-day compressive strength; (**b**) 28-day modulus of elasticity; (**c**) 28-day splitting tensile strength; (**d**) 28-day flexural strength.

The relationships between the different 28-day concrete properties are analyzed in Figure 8 and Figure 9 by considering the average values of the concrete properties. Figure 8 considers compressive strength as the estimating variable, following the traditional tendency of the standards to predict all mechanical properties as a function of that strength [40]. On the other hand, Figure 9 considers UPV as the independent variable, since this is a non-destructive property that has traditionally been used as an indirect measure of concrete quality and, therefore, of its mechanical behavior [55,56]. The low number of mixtures examined leads this analysis to be preliminary. It was not intended to develop broadly valid models to estimate the mechanical properties of concrete with SCWTB. The objective was to define which concrete property should be considered as independent variable to develop more comprehensive models to predict all the mechanical properties of concrete containing SCWTB in future research.

The elastic modulus was the mechanical property that could be most accurately estimated with compressive strength (Figure 8a), reaching a linear regression R^2^ coefficient of almost 99%. Both mechanical properties characterize the behavior of concrete under compression [58,59], so they are closely related and followed similar trends, as observed in this research (Figure 4 and Figure 5a). The splitting tensile strength estimation (Figure 8b) was less accurate (R^2^ coefficient of 66.2%) because of the stitching effect of the GFRP composite fibers in the MS3.0 mix. However, the effect of the fibers was more appreciable on flexural strength, so it followed a completely different trend to that of the compressive strength, the relationship between both being practically non-existent (Figure 8c), reaching only an R^2^ coefficient of 1.7%.

Mechanical properties were estimated less accurately on the basis of the UPV readings than on the basis of compressive strength, which was perhaps due to the small variations in this non-destructive measure compared to those of the mechanical properties when the SCWTB amount in the concrete was modified [37,56]. The properties that were more accurately estimated were those related to the behavior under compression, especially the modulus of elasticity (R^2^ coefficient of 81.1%, Figure 9b), a variable that significantly conditions the UPV of concrete [55]. The linear regression of splitting tensile strength (Figure 9c) and flexural strength (Figure 9d) had R^2^ coefficients of only 51.2% and 11.2%, respectively. UPV does not adequately represent the fiber stitching effect within the cementitious matrix [48], apart from the fact that the bending tensile mechanical properties of certain types and compositions of concrete are not adequately estimated using UPV, according to the literature [60].

**Figure 10:**
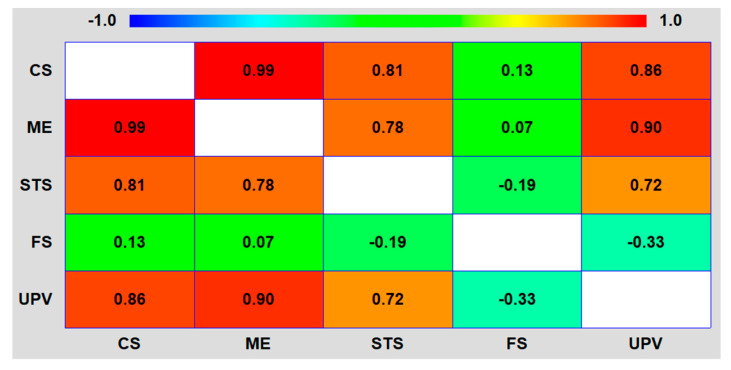
Pearson’s correlation matrix (legend: CS, compressive strength; ME, modulus of elasticity; STS, splitting tensile strength; FS, flexural strength; UPV, ultrasonic pulse velocity).

To reduce biases and to corroborate all aspects detected through the analysis of property relationships, a second analysis was conducted by calculating the Pearson’s correlations (Figure 10). The compressive strength showed correlations with the modulus of elasticity and the splitting tensile strength closer to 1 than the UPV and therefore more accurate relationships with them [60]. In addition, both compressive strength and UPV exhibited a very low correlation value with flexural strength, being therefore both properties inadequate to predict the flexural strength of the concrete with SCWTB [21].

### 3.8. Significance of the SCWTB Content

The significance of the SCWTB content of concrete on its mechanical properties was evaluated by considering all the individual experimental results through a one-way analysis of variance (ANOVA) at a 95% confidence level. The factor analyzed was the SCWTB amount. Table 5 shows the *p*-values for each property as well as the homogeneous groups, i.e., those SCWTB contents that resulted in statistically equal values for a certain concrete property and that did not exhibit variability between them [60]. The results showed the validity of the SCWTB contents considered to define the effect of SCWTB on the mechanical performance of concrete.

According to the *p*-values, changes in the air content, the Poisson’s coefficient, and the UPV readings were not linked to the amounts of SCWTB in the concrete samples. The variation of those properties was very small with the addition of SCWTB, as highlighted in previous sections, which explains these statistical results [61]. In relation to the other properties, in which the SCWTB content did significantly affect concrete performance, the results reinforced some of the above-mentioned aspects:

- The addition of 1.5% and 3.0% SCWTB did not lead to a relevant variation in density, either in the fresh or in the hardened state.
- MS3.0 and MS4.5 mixes always presented statistically identical behavior under compression, i.e., regarding their compressive strengths and moduli of elasticity. At advanced ages, their behavior was also equivalent to that of the MS1.5 mix. Therefore, only the addition of high SCWTB contents, 6.0% in this study, significantly affected the compressive behavior of concrete, with the GFRP composite fiber stitching effect performing well within the cementitious matrix [51].
- When pure tensile stresses were applied, the MS1.5 and MS3.0 mixes, on the one hand, and the MS4.5 and MS6.0 mixes, on the other, showed equivalent behavior. The bridging effect of the GFRP composite fibers in the SCWTB was effective mainly at small contents, due to the notable negative impact of the increase of the water proportion [48]. Nevertheless, the statistical performance of both the MS1.5 and the MS6.0 mixes was virtually equal in terms of flexural strength. Thus, the addition of SCWTB in large quantities led to a higher increase in the GFRP composite fibers that were more effective at stitching the cementitious matrix to resist bending stresses [21].

### 3.9. Micro-Structural Analysis

**Figure 11:**
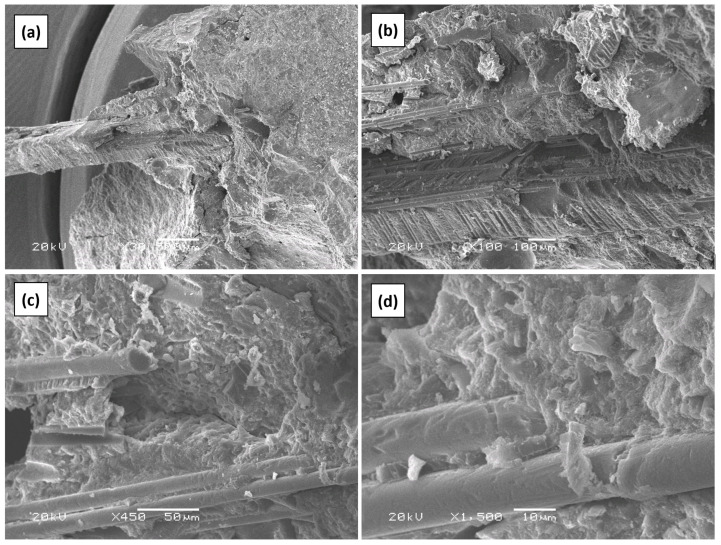
SEM analysis of the interfacial transition zone between a GFRP composite fiber and the cementitious matrix in the MS6.0 mix: (**a**) 30× magnification; (**b**) 100× magnification; (**c**) 450× magnification; (**d**) 1500× magnification.

As the ANOVA showed, the GFRP composite fibers present in the SCWTB caused the mechanical performance of the mixtures to be preserved when the waste was added, despite the increased water amount. This behavior was indicative of adequate adhesion within the cementitious matrix of the GFRP composite fibers, which implied an effective fiber stitching effect [52,57]. An SEM analysis was performed on fragments of specimens of the MS6.0 mix tested at 90-day compressive strength for further investigation and verification of that property. The main images of the analysis are depicted in Figure 11.

The SEM analysis verified the aspects revealed by the trends followed by the mechanical properties. It was found that the GFRP composite fibers of larger size were optimally embedded within the cementitious matrix (Figure 11a), so they did not become detached when handling the concrete specimens for the mechanical behavior tests, ensuring successful stitching of cracks within the cementitious matrix [48]. This good adhesion could have been favored by the rough surface texture provided by the epoxy resin matrix (Figure 11b). The same performance was also observed in the micro-fibers at 450× magnification (Figure 11c), with no abrupt boundary between both materials detected, even at 1500× magnification (Figure 11d).

### 3.10. Comparison Between Waste Types

As highlighted above, the concrete mixes in this investigation and in previous research by the same authors had exactly the same design and composition [37]. The only difference between them was the type of wind turbine blade waste. The mixes of the previous research incorporated RCWTB, which not only consisted of GFRP composite fibers, but also large percentages of polymer and balsa wood particles [35]. The research presented in this article was conducted with SCWTB, which consisted almost exclusively of GFRP composite fibers, along with minimal quantities of the remaining blade components. Those differences can be noted in Table 6. However, the same table also shows that both types of wind turbine blade wastes were almost identical in terms of fiber content, as well as in terms of dimensional and strength characteristics of the fibers. This situation was because the crushing process of the pieces of wind turbine blades was identical in both cases, which guaranteed that the concretes produced with both waste types were fully comparable [1,2].

Table 7 shows a comparison for each waste content between the properties of concrete containing SCWTB, presented throughout this paper, and those of concrete containing RCWTB, which can be found in an author’s previous paper [37]. This table shows the percentage variation between the properties of the concretes containing both waste types calculated with Equation (1), taking as a reference the concrete containing RCWTB. Thus, positive values indicated that SCWTB led to a better performance, while negative values were indicative that the behavior of RCWTB concrete was better. In addition, the table also displays the *p*-value of a two-way ANOVA to determine the significance of the type of wind turbine blade waste.

(1) $$V=\frac{P_{SCWTB}-P_{RCWTB}}{P_{RCWTB}}\times 100$$

The waste type was not significant in three aspects: both fresh and hardened density, UPV, and flexural strength. Both waste types presented a lower density than the rest of the components of the concrete mix, so they led to a similar density drop. UPV is closely related to density [55], so it replicated that same behavior. Finally, flexural strength also did not depend on the waste type. The proportion of fibers was the same in both waste types, which also showed almost identical characteristics (Table 6). The cementitious matrix was therefore stitched with the same efficiency in both cases, which meant that the balsa wood and polymeric particles did not have an especially negative effect. These results could simplify the recycling process of wind turbine blades when yielding a by-product to be used as a raw material in concrete intended to work under bending stresses, as no separation of the blade’s components would be needed prior to crushing. These particles did have a negative effect on other properties, as their absence in the SCWTB generally led to higher slumps, higher compressive and splitting tensile strengths, and higher elastic moduli. However, these particles limited the transverse deformability of the concrete specimens, an aspect evaluated with Poisson’s coefficient [40]. Therefore, the addition of SCWTB to concrete would be recommended for applications where the concrete element works mainly under compression stresses.

## 4. Conclusions

The in-fresh and the mechanical behavior of concrete produced with selectively crushed wind turbine blade (SCWTB) has been evaluated in this research. Wind turbine blade pieces composed almost exclusively of glass fiber-reinforced polymer (GFRP) were crushed for SCWTB production, so GFRP composite fibers were therefore almost the sole component of this waste. SCWTB contents of 1.5%, 3.0%, 4.5%, and 6.0% were added to the concrete. The following conclusions can be drawn from the discussions throughout the paper:

- Despite the increase of the content of plasticizers and water, higher SCWTB contents slightly reduced the concrete slump, because of the irregular shape of the GFRP composite fibers and their high specific surface area that had to be covered by a water film. Air content was not affected by SCWTB additions.
- The low density of SCWTB, with a value of 1.50 kg/dm^3^, resulted in a decrease of both hardened and fresh concrete density. Ultrasonic pulse velocity (UPV) was fundamentally conditioned by density, so this property also decreased with increasing SCWTB contents. Nevertheless, UPV measurements were in accordance with a good quality concrete in all cases.
- The compressive strength and elastic modulus were reduced at early ages as the SCWTB content increased, a reduction that was only observed at later ages for high SCWTB contents (6.0%). Intermediate SCWTB contents (3.0% and 4.5%) led to similar concrete compression behavior at all ages. Finally, the long-term temporal strength development was reduced only by 6.0% SCWTB. The stitching of the concrete matrix by the GFRP composite fibers was not effective when applying compression stresses, the increase in the water/cement ratio when adding SCWTB being more relevant.
- The GFRP composite fibers in SCWTB showed proper adhesion to the concrete matrix, as scanning electron microscopy demonstrated, which allowed them to exhibit an effective stitching effect. This phenomenon was verified in different mechanical properties. Thus, despite the increasing water/cement ratio with growing SCWTB contents, the Poisson’s coefficient remained constant from 3.0% SCWTB, the splitting tensile strength was maintained up to 3.0% SCWTB, and the mixes with 1.5% and 6.0% SCWTB demonstrated very similar flexural strengths.
- Compressive strength and UPV showed the most precise relationship with the elastic modulus, which was a little affected by the stitching effect of the GFRP composite fibers. The relationship was always more precise when compressive strength was considered.

Overall, the behavior of all the mechanical properties of the concrete samples containing up to 6.0% SCWTB was adequate. Furthermore, SCWTB concrete showed better workability, compression behavior, and splitting tensile strength than that the concrete made with raw-crushed wind turbine blade [37], which incorporated not only GFRP composite fibers, but also near-spherical particles of balsa wood and polymers [35]. However, the flexural strength of concrete was the same when using both types of wind turbine blade waste, as both wastes contained almost identical fiber proportions of almost the same dimensions and strength characteristics. Based on these results, future research could address optimum designs of SCWTB mixes, and evaluate their performance in terms of mechanical performance, durability, sustainability and cost. From them, the most appropriate SCWTB content in concrete could be defined from a multi-criteria approach.

## Figures and Tables

**Table 1:** Physical properties of SCWTB: average values and standard deviations.

Property	Reference Standard [38,44]	Unit	Value
Content of fibers	EN 933-11	wt.%	62.6 ± 2.9
Content of micro-fibers	EN 933-11	wt.%	35.5 ± 2.1
Content of other components	EN 933-11	wt.%	1.9 ± 0.1
Overall density	EN 1097-6	kg/dm^3^	1.50 ± 0.02
Apparent density	EN 1097-3	kg/m^3^	84.3 ± 3.7
Length of fibers	ASTM D5103	mm	14.2 ± 4.48
Equivalent diameter of fibers	ASTM D2130	mm	0.75 ± 0.13
Tensile strength of fibers	EN ISO 6892-1	MPa	279 ± 5

**Table 2:** Mix design.

Property	Comparative (kg) ^1^				kg/m^3 2^			
	MS1.5	MS3.0	MS4.5	MS6.0	MS1.5	MS3.0	MS4.5	MS6.0
Cement	320	320	320	320	312	306	300	295
Coarse gravel 6/22 mm	900	900	900	900	878	861	845	829
Fine gravel 2/6 mm	600	600	600	600	585	574	563	553
Sand 0/2 mm	500	500	500	500	488	478	469	461
Water	133	137	142	146	129	131	133	134
Plasticizer 1	2.62	3.04	3.46	3.88	2.56	2.91	3.25	3.57
Plasticizer 2	1.31	1.52	1.73	1.94	1.28	1.45	1.62	1.79
SCWTB	22.5	45.0	67.5	90.0	21.9	43.0	63.4	82.9
Mix volume (L)	1026	1045	1066	1086	1000	1000	1000	1000

^1^ Comparative mix compositions appear under the column heading “Comparative (kg)”. ^2^ Concrete mix compositions per cubic meter appear under the column heading “kg/m^3^”.

**Table 3:** Experimental plan.

Standard [38]	Test	Specimen Shape	Specimen Dimensions (mm)	Age (Days)
EN 12350-2	Slump	Fresh concrete sample	-	Fresh
EN 12350-6	Fresh density	Fresh concrete sample	-	Fresh
EN 12350-7	Air content	Fresh concrete sample	-	Fresh
EN 12390-7	Hardened density	Cubic	100 × 100 × 100	28
EN 12390-3	Compressive strength	Cylindrical	100 × 200	7, 28, 90
EN 12390-13	Modulus of elasticity	Cylindrical	100 × 200	28, 90
EN 12390-13	Poisson’s coefficient	Cylindrical	100 × 200	28
EN 12504-4	Ultrasonic pulse velocity (UPV)	Cubic	100 × 100 × 100	28, 90
EN 12390-6	Splitting tensile strength	Cylindrical	100 × 200	28
EN 12390-5	Flexural strength	Prismatic	75 × 75 × 275	28

**Table 4:** Fresh properties of the concrete mixes.

Mix	Slump (cm)	Fresh Density (kg/dm^3^)	Air Content (% vol.)
MS1.5	15.0 ± 0.5	2.36 ± 0.03	2.4 ± 0.2
MS3.0	14.5 ± 0.5	2.36 ± 0.02	2.4 ± 0.1
MS4.5	12.0 ± 0.5	2.32 ± 0.02	2.7 ± 0.2
MS6.0	11.0 ± 0.0	2.27± 0.02	2.5 ± 0.1

**Table 5:** ANOVA with a confidence level of 95%.

Property	*p*-Value for SCWTB Content	Homogeneous Groups
Slump	0.0000	-
Fresh density	0.0039	MS1.5 and MS3.0
Air content	0.2564	All mixes
Hardened density	0.0074	MS1.5 and MS3.0
7-day compressive strength	0.1780	All mixes
28-day compressive strength	0.0117	MS3.0 and MS4.5
90-day compressive strength	0.0197	MS1.5, MS3.0, and MS4.5
28-day modulus of elasticity	0.0308	MS3.0 and MS4.5
90-day modulus of elasticity	0.0287	MS1.5, MS3.0, and MS4.5
28-day Poisson’s coefficient	0.8408	All mixes
28-day ultrasonic pulse velocity	0.2071	All mixes
90-day ultrasonic pulse velocity	0.4781	All mixes
28-day splitting tensile strength	0.0291	MS1.5 and MS3.0; MS4.5 and MS6.0
28-day flexural strength	0.0101	MS3.0 and MS4.5; MS1.5 and MS6.0

**Table 6:** Comparison of physical properties of SCWTB (Table 1) and RCWTB [35].

Property	SCWTB	RCWTB
Overall density (kg/dm^3^)	1.50	1.63
Apparent density (kg/m^3^)	84.3	246.6
Content of fibers (% wt.)	62.6	66.8
Content of micro-fibers (% wt.)	35.5	13.8
Content of other components (% wt.)	1.9	19.4
Length of fibers (mm)	14.2	13.1
Equivalent diameter of fibers (mm)	0.75	0.73
Tensile strength of fibers (MPa)	279	270

**Table 7:** Comparison between SCWTB and RCWTB mixes.

Property	Variation (%)				*p*-Value for Waste Type *
	1.5% Waste	3.0% Waste	4.5% Waste	6.0% Waste	
Slump	42.9	11.5	−11.1	−8.3	0.0000
Fresh density	−0.4	0.4	0.9	−0.4	0.9454
Air content	20.0	20.0	22.7	−3.8	0.0000
Hardened density	0.4	0.4	0.9	−0.4	0.8373
7-day compressive strength	−6.1	4.7	27.0	20.8	0.0000
28-day compressive strength	−4.0	1.7	17.3	9.3	0.0000
90-day compressive strength	−11.8	0.2	10.7	−0.4	0.0000
28-day modulus of elasticity	9.3	10.4	32.5	12.4	0.0000
90-day modulus of elasticity	9.5	14.6	35.8	14.2	0.0000
28-day Poisson’s coefficient	−22.9	−12.6	−12.6	−12.7	0.0000
28-day ultrasonic pulse velocity	1.6	1.4	1.7	−3.7	0.7438
90-day ultrasonic pulse velocity	−0.5	−2.9	−0.9	−3.6	0.0511
28-day splitting tensile strength	16.2	19.5	29.5	32.0	0.0000
28-day flexural strength	4.1	−0.2	−0.6	0.8	0.1159

* *p*-values calculated according to a two-way ANOVA with a confidence level of 95%.

## Data Availability

The original contributions presented in this study are included in the article. Further inquiries can be directed to the corresponding author.
